# Trends in Incidence Rates during 1999-2008 and Prevalence in 2008 of Childhood Type 1 Diabetes Mellitus in GERMANY – Model-Based National Estimates

**DOI:** 10.1371/journal.pone.0132716

**Published:** 2015-07-16

**Authors:** Alexander Bendas, Ulrike Rothe, Wieland Kiess, Thomas Michael Kapellen, Thoralf Stange, Ulf Manuwald, Eckhard Salzsieder, Reinhard Walter Holl, Olaf Schoffer, Anna Stahl-Pehe, Guido Giani, Stefan Ehehalt, Andreas Neu, Joachim Rosenbauer

**Affiliations:** 1 Department of Otorhinolaryngology, University Hospital Dresden, Dresden, Germany; 2 Health Sciences/Public Health, Faculty of Medicine “Carl Gustav Carus”, TU Dresden, Dresden, Germany; 3 Hospital for Children and Adolescents, Center for Pediatric Research, Department of Women and Child Health, University of Leipzig, Leipzig, Germany; 4 Institute for Medical Informatics and Biometry, Faculty of Medicine “Carl Gustav Carus”, TU Dresden, Dresden, Germany; 5 Institute of Diabetes “Gerhardt Katsch” Karlsburg, Karlsburg, Germany; 6 Institute for Epidemiology and Medical Biometry, University of Ulm, Ulm, Germany; 7 Department of Tumorepidemiology, Faculty of Medicine “Carl Gustav Carus”, TU Dresden, Dresden, Germany; 8 Institute for Biometrics and Epidemiology, German Diabetes Centre, Leibniz Institute for Diabetes Research at Heinrich Heine University Düsseldorf, Düsseldorf, Germany; 9 Public Health Department of Stuttgart, Department of Pediatrics, Dental Health Care, Health Promotion and Social Services, Stuttgart, Germany; 10 University Children’s Hospital Tübingen, Tübingen, Germany; Indiana University School of Medicine, UNITED STATES

## Abstract

**Aims:**

To estimate the national incidence rate and trend of type 1 diabetes (T1DM) in Germany from 1999 to 2008 and the national prevalence in 2008 in the age group 0–14 years.

**Methods:**

Data were taken from a nationwide registry for incident cases of T1DM in the ages 0–4 years and 3 regional registries (North-Rhine-Westphalia, Baden-Wuerttemberg and Saxony) for incident cases of T1DM in the ages 0–14 years covering 41% of the child population in Germany. The degree of ascertainment was ≥ 97% in all registries. Incident and prevalent cases were grouped by region, sex, age (0–4, 5–9, 10–14 years), and, for incident data, additionally by two 5-year periods (1999–2003, 2004–2008). Poisson regression models were fitted to the data to derive national estimates of incidence rate trends and prevalence in the age groups 5–9, 10–14 and 0–14 years. We used direct age-standardization.

**Results:**

The estimated national incidence rate in 0-14-year-olds increased significantly by 18.1% (95%CI: 11.6–25.0%, p<0.001) from 1999–2003 to 2004–2008, independent of sex, corresponding to an average annual increase of 3.4% (95%-CI: 2.2–4.6%). The overall incidence rate was estimated at 22.9 per 100,000 person-years and we identified a within-country west-east-gradient previously unknown. The national prevalence in the ages 0–14 years on 31/12/2008 was estimated to be 148.1 per 100,000 persons.

**Conclusions:**

The national incidence rate of childhood T1DM in Germany is higher than in many other countries around the world. Importantly, the estimated trend of the incidence rate confirms the international data of a global increase of T1DM incidences.

## Introduction

Today, type 1 diabetes mellitus (T1DM) is considered an autoimmune disease with a T-cell mediated destruction of the insulin producing beta cells in the pancreas. In addition to genetic factors, environmental exposures are thought to play an important role in the etiology of the disease, possibly in interplay with genetic factors. However, the underlying mechanisms are largely unknown [1].

The worldwide incidence rate of T1DM in the age group 0–14 years varies considerably depending on the region. The range is from 1 to 58 per 100,000 person-years [2–7]. Likewise, the predicted cases of the prevalence of T1DM in this age group ranged between 20 and 500 per 100.000 in Europe in 2010 [8].

Current national data of the incidence rate in children and adolescents (0–14 years) are not available for Germany because there is no nation-wide registration of T1DM cases in this age group. Previous estimates of T1DM incidence rates in this age group from regional German registries showed regional variation [5] meaning that simple extrapolation to the national level could be associated with considerable bias.

National data of the incidence rate in the age group 0–14 years could only be registered in the former East Germany and only through 1989 [9]. In Germany as a whole, however, a national incidence registry (Erhebungseinheit für seltene pädiatrische Erkrankungen in Deutschland, ESPED) [10] has only been available for children from 0 to 4 years since 1993, but there are 3 operating regional registries [11–13] for incident cases of T1DM in the ages 0–14 years, all of which participate in the EURODIAB project (European Community Concerted Action Programme on the Epidemiology and Prevention of Diabetes) [5, 6].

The objective of this paper is to use these age-limited (0–4 years) national data (ESPED) in connection with available regional data for older ages to provide better estimates of the national childhood T1DM risk for the age group 0–14 years [14]. Therefore, we aimed to provide estimates of the national incidence rate of childhood T1DM in Germany, its trend over the observation period, and the prevalence of childhood T1DM by a model-based approach merging all available regional (0–14 years) and national (0–4 years) data on childhood T1DM.

## Methods

### Data Sources

For this study, we pooled anonymized data from a nationwide registry for incident cases of T1DM in the ages 0–4 years (German registry (ESPED), maintained at the German Diabetes Centre (DDZ)) and 3 regional registries for incident cases of T1DM in the ages 0–14 years covering the federal states of North Rhine-Westphalia (NW), Baden-Wuerttemberg (BW) and Saxony (SN) [10–17]. The 3 regional registries comprise 41% of the total child population aged 0–14 years in Germany. All 4 registries have been collecting incident cases of new-onset childhood T1DM prospectively since the 1990s based on EURODIAB criteria [5, 6]. As recommended, secondary sources are used to complement the primary case ascertainment. Details of the 4 registries in particular on primary and secondary data sources and estimated completeness of ascertainment are summarized in Table 1. For this analysis, incident cases of the 10-year period 1999–2008 and prevalent cases on 31/12/2008 were extracted from the 4 registries.

**Table 1:** Characteristics of type 1 diabetes registries in Germany used for the estimation of incidence rates and prevalence.

Registry	Region covered	Age range (years)	Start of prospective registration	Primary sources	Secondary sources	Estimated completeness
German registry [10, 11, 14]	Germany	0–4	1993	Hospital-based active surveillance system (ESPED*)	Annual surveys among medical practices (only NW), DPV database	97%
North Rhine-Westphalian (NW) registry [5, 11, 14, 17]	North-Rhine-Westphalia	0–14	1996	Hospital-based active surveillance system (ESPED)	Annual surveys among medical practices, DPV database	99%
	Düsseldorf region	0–14	1993 (1987–1992 retrospective)	Hospital-based active surveillance system (ESPED)	Annual surveys among medical practices, DPV database	99%
Baden-Wuerttemberg (BW) Diabetes Incidence Registry (DIARY) [5, 12, 14–16]	Baden-Wuerttemberg	0–14	1997 (1987–1996 retrospective)	Hospital based registration system	Surveys among participants of state-wide diabetes information events	98%
Childhood Diabetes Registry Saxony (SN) [5, 13] [18]	Saxony	0–14	1999 (1998 retrospective)	Hospital based registration system, (DPV database additionally)	Public health school examinations at ages 6, 11 and 15 years	94% (2004); 97% (2008)

*ESPED: **E**rhebungseinheit für **s**eltene **p**ädiatrische **E**rkrankungen

DPV: **D**iabetesprogramm zur **p**rospektiven **V**erlaufsbeobachtung (computer-based longitudinal documentation of therapy and outcomes in diabetes car for quality control and diabetes research)

**Fig 1:**
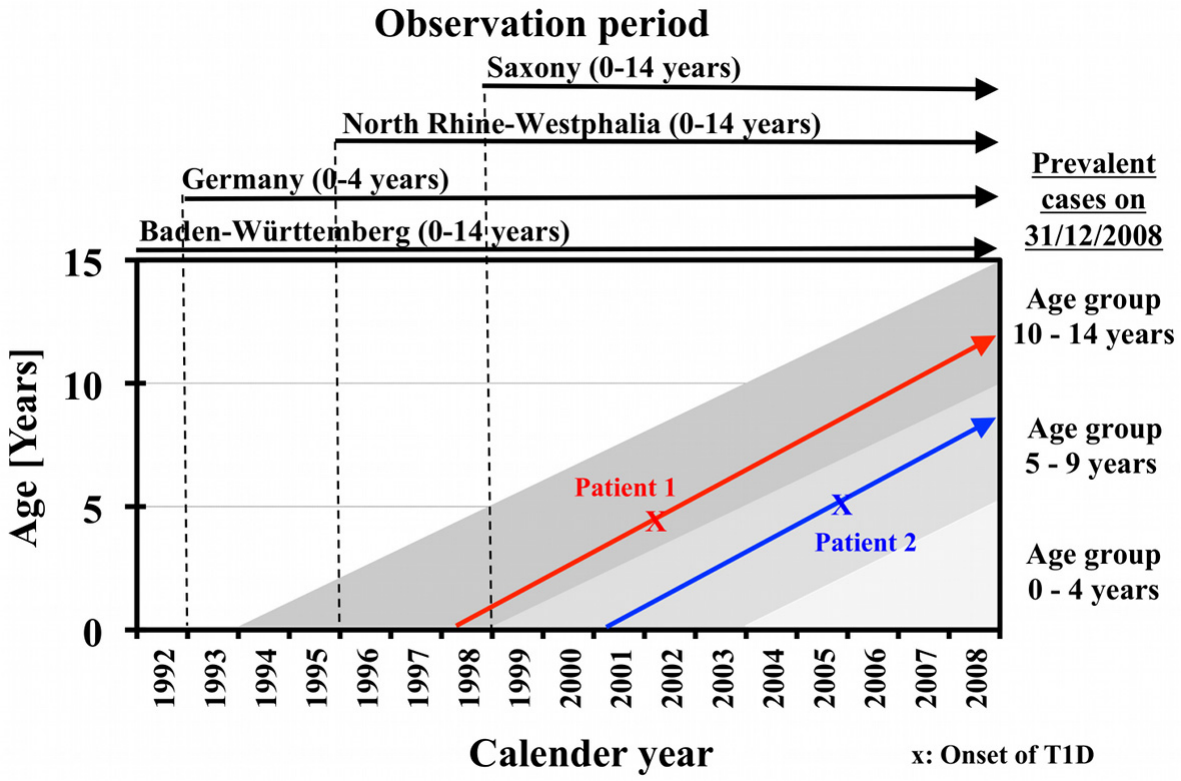
Prevalent cases on 31/12/2008 – Lexis diagram.

Prevalent cases in the ages 0–14 years on 31/12/2008 correspond to all incident cases of the 3 regional registries covered the by the grey shaded area of the Lexis diagram in Fig 1. Incident cases of the grey shaded area in the period 1999–2008 are covered by the 3 regional registries, which registered all incident cases in the ages 0–14 years during this period (Table 1). Incident cases–if less than 5 years of age at onset–in the grey shaded area from the period 1994–1998 are covered by the national registry for 0–4 year-olds. Therefore, all prevalent cases for those 0–14 years of age on 31/12/2008 in the 3 federal states are potentially covered by the 4 incidence registries used.

For analysis, incident cases were grouped by registry/region (NW, BW, SN, Germany), sex, age at onset (0–4, 5–9, 10–14 years), and period (1999–2003, 2004–2008). Prevalent cases were grouped by registry/region (NW, BW, SN, Germany), sex (male, female), and age on 31/12/2008 (0–4, 5–9, 10–14 years).Population data were obtained from the Federal Statistical Office [19].

The data protection agencies in SN, NW and BW approved the registries and this analysis.

### Ethics Statement

This article does not contain any studies with human or animal subjects performed by any of the authors.

### Statistical Analysis

Based on registered incident cases, age- and sex-specific incidence rates and respective confidence intervals (95%-CIs) were estimated using the person-time method (incident cases/person-time) assuming a Poisson distribution of cases. Sex-specific estimates of the incidence rate for the total age group 0–14 years were age-standardized according to the direct method using equal weights for age groups. Further, cumulative incidences were estimated from age-specific incidence rates as cumulative hazard functions according to standard methods [20]. CIs for standardized and cumulative incidence rates were estimated using the normal approximation. Incidence rates are presented per 100,000 person-years (py) and cumulative incidences (with CIs) per 100,000. The increase in the incidence rate was estimated from rate ratios of 5-year period incidence rates. Age- and sex-specific prevalence and age-standardized prevalence were estimated based on prevalent cases (prevalent cases/population) and are presented per 100,000 persons.

In order to derive estimates of the national incidence rates from registered incident cases, we fitted several Poisson regression models to incidence data stratified by region, period and age taking into account second and third order interaction terms of these as independent variables. Likewise, we fitted several Poisson regression models to incidence data additionally stratified by region, period, sex, age, and again taking into account second- and higher order interaction terms. In order to avoid over fitting and to select parsimonious models, the Bayesian information criterion [21] was applied in model selection. Poisson models with best (lowest) Bayesian information were selected to calculate model-based estimates of the incidence rates. If assuming rate ratios between age groups to be independent of region the selected models allowed for estimation of incidence rates figures even for subgroups without observed cases, stratified by period and sex, i.e. for age groups 5–9 and 10–14 years at the national level. With this approach, the national incidence rates in age groups 5–9 and 10–14 years were in fact estimated by applying the model-based incidence rate ratios estimated between age groups from the regional data of the 3 federal states to the national level. National estimates for the total cohort covering 0–14 years were derived from age-specific model-based estimates by direct age-standardization using equal weights for age groups. In addition, cumulative incidences were estimated from age-specific figures. Increases in incidence rates between both 5-year periods estimated from Poisson models were transformed to annual increases by taking the 5^th^ root of these numbers.

We used the same Poisson regression approach to derive estimates of the national prevalence from observed prevalent cases. We fitted Poisson regression models to prevalence data stratified by region and age with region, age, and a region by age interaction as independent variables. Further, we fitted several Poisson regression models to prevalence data additionally stratified by sex with region, sex, age, and second- and third order interaction terms of these as independent variables. Again, Poisson models with best (lowest) Bayesian information criterion were selected to calculate model-based estimates of the prevalence.

In all models, over-/under dispersion of incident/prevalent cases was taken into account by introducing a multiplicative dispersion factor in the variance function [22] (“dscale” option in SAS “genmod” procedure). The goodness of fit of models selected to estimate national figures was assessed by pseudo-R^2^ [23]. Within Poisson models, groups were compared with likelihood ratio or Wald χ^2^-tests. Two-sided p-values ≤ 0.05 were considered statistically significant. All analyses were performed with SAS 9.3 (Statistical Analysis Software, SAS Institute Inc., Cary, NC, USA).

## Results

### Estimates Based on Observed Cases

#### Incidence rate

The age-standardized incidences rates varied by region and period. Overall, incidence rates were higher in North Rhine-Westphalia (1999–2003: 21.3 (95%CI 20.5–22.0), 2004–2008: 24.4 (95%CI 23.5–25.2)) and Baden-Wuerttemberg (1999–2003: 17.0 (95%CI 16.1–17.8), 2004–2008: 22.3 (95%CI 21.3–23.3)) (both in West Germany) than in Saxony (1999–2003: 15.6 (95%CI 14.1–17.2), 2004–2008: 20.3 (95%CI 18.5–22.3)) (former Eastern Germany). Between the periods of 1999–2003 and 2004–2008 the age-standardized incidence rates increased in North Rhine-Westphalia by 14.6%, in Baden-Wuerttemberg by 31.2% and in Saxony by 30.1%.

#### Cumulative incidence

In the period 1999–2003, the cumulative incidence at the age of 15 years in the 3 regions ranged from 243.1 to 324.4 per 100,000 males and 223.7 to 313.2 per 100,000 females. In the period 2004–2008 the corresponding figures are 325.2 to 371.8 per 100,000 males and 284.3 to 358.6 per 100,000 females. The increases in the cumulative incidence corresponded to the increases in the age-standardized incidence rates.

#### Prevalence

The age-standardized prevalence varied by region. By 31.12.2008, the prevalence was 150.7 (95%CI 146.1–155.4) per 100.000 persons in North Rhine-Westphalia, 132.6 (95%CI 127.2–138.3) in Baden-Wuerttemberg and 129.1 (95%CI 118.7–140.4) in Saxony.

### Model-Based Estimates

#### Incidence rate

We fitted several Poisson models to incidence data stratified by region, period and age group (data not shown). According to the Bayesian information criterion, the best fitting model (Poisson model M1) included region, period, age, and a term for region by period interaction as independent predictors.

Estimates of the incidence by region, period and age predicted from this best Poisson model are given in Table 2. The predicted estimates were in good agreement with the observed incidence rates (pseudo-R^2^ = 0.978).

**Table 2:** Incidence rates and cumulative incidence of T1DM by region, period and age predicted from Poisson model M1.

				Incidence rates (95% CI) per 100,000 person-years				Cumulative incidence (95% CI) per 100,000
Region	Period	N	Person-years	0–4 years	5–9 years	10–14 years	0–14 years*	15 years
**North Rhine-Westphalia**	1999–2003	3,112	14,476,032	15.9 (15.1–16.8)	23.5 (22.5–24.6)	24.4 (23.3–25.5)	21.3 (20.7–21.9)	319.2 (310.8–327.9)
	2004–2008	3,295	13,317,041	18.2 (17.3–19.2)	27.0 (25.8–28.2)	28.0 (26.8–29.2)	24.4 (23.8–25.0)	365.9 (356.3–375.6)
**Baden-Wuerttemberg**	1999–2003	1,492	8,729,465	12.7 (11.9–13.5)	18.8 (17.7–19.9)	19.4 (18.3–20.6)	17.0 (16.4–17.6)	254.3 (245.6–263.4)
	2004–2008	1,832	6,570,440	16.6 (15.6–17.6)	24.6 (23.3–25.9)	25.4 (24.1–26.8)	22.2 (21.5–22.9)	333.0 (322.4–344.0)
**Saxony**	1999–2003	411	2,615,993	11.5 (10.3–12.8)	17.0 (15.3–18.9)	17.7 (15.9–19.6)	15.4 (14.5–16.4)	231.1 (217.3–245.7)
	2004–2008	445	2,224,385	15.2 (13.7–16.8)	22.4 (20.3–24.8)	23.2 (21.0–25.7)	20.3 (19.1–21.5)	304.1 (286.7–322.7)
**Germany**	1999–2003	12,335	62,870,790	14.5 (14.0–15.1)	21.5 (20.1–22.9)	22.2 (20.8–23.7)	19.4 (18.7–20.1)	291.1 (280.9–301.8)
	2004–2008	13,299	57,436,698	17.1 (16.5–17.8)	25.4 (23.8–27.1)	26.3 (24.6–28.0)	22.9 (22.1–23.7)	343.8 (331.8–356.2)

*age-standardized, Poisson model M1 included region, period, age at onset (0.4, 5–9, 10–14 years) and a term for region by period interaction as independent variables, pseudo-R^2^ = 0.978

Predicted national estimates of the incidence rate are also given in Table 2. The model-based national incidence rate in the cohort of 0–14 year-olds increased significantly between the two periods (p<0.001; Table 2). According to the best Poisson model, the national incidence rate increased independently of age by 18.1% (95% CI: 11.6–25.0%), corresponding to an average annual increase of 3.4% (95% CI: 2.2–4.6) in Germany.

The model-based increase in incidence rates varied significantly (p<0.001) between regions. In North Rhine-Westphalia the increase was 14.6% (95% CI: 8.6–21.0), in Baden-Wuerttemberg 30.9% (95% CI: 21.4–41.2), and in Saxony 31.6% (95% CI: 13.5–53.7). The corresponding annual increases were 2.8% (95% CI: 1.7–3.9), 5.5% (95% CI: 4.0–7.1), and 5.7% (95% CI: 2.6–8.8), respectively.

In both periods, the estimated national incidence rate and the incidence rate in North Rhine-Westphalia were significantly higher than the incidence rate in Saxony (p<0.001).

The best fitting model for incidence data stratified by region, period, sex and age group (Poisson model M2) included region, period, sex, age group, and terms for region by period and sex by age group interactions as independent predictors. Sex-specific, age-standardized national and regional estimates of the incidence rate derived from this Poisson model are given in Table 3.

**Table 3:** Age-standardized incidence rates by region, period and sex predicted from Poisson model M2.

		Incidence rate* (95% CI) per 100,000 person-years	
Region	Period	Male	Female
**North Rhine-Westphalia**	1999–2003	21.8 (21.1–22.5)	20.8 (20.0–21.5)
	2004–2008	25.0 (24.1–25.8)	23.8 (23.0–24.6)
**Baden-Wuerttemberg**	1999–2003	17.4 (16.6–18.1)	16.5 (15.9–17.2)
	2004–2008	22.7 (21.9–23.6)	21.7 (20.8–22.5)
**Saxony**	1999–2003	15.8 (14.7–16.9)	15.0 (14.0–16.1)
	2004–2008	20.8 (19.4–22.2)	19.8 (18.5–21.1)
**Germany**	1999–2003	19.9 (19.0–20.7)	18.9 (18.1–19.8)
	2004–2008	23.5 (22.5–24.5)	22.4 (21.4–23.3)

*age-standardized; population 0–14 years, Poisson model M2 included region, period, sex, age at onset (0–4, 5–9, 10–14 years) and terms for age at onset by sex and region by period and interactions as independent variables, pseudo-R^2^ = 0.953

#### Cumulative incidence

Model-based cumulative incidence estimates at the age of 15 years were derived from Poisson model M1. For each region, period and age group, the predicted estimates were in good agreement with the observed cumulative incidences from the 3 regional registries (Table 2). Predicted national cumulative incidence estimates are also given in Table 2. The estimates of the national cumulative incidences for the period 2004–2008 imply that 1 out of 291 (95% CI 281–301) new-borns will develop T1DM before the age of 15 years. The model-based increases in the cumulative incidence correspond to the increases in the incidence, both nationwide and for the 3 regions.

Model-based estimates of sex-specific cumulative incidences were derived from the Poisson model M2 and were also in good agreement with the observed cumulative incidences from the 3 regional registries (data not shown). The estimate of the national cumulative incidence for the period 1999–2003 was 298.0 (285.7–310.8) among males and 284.0 (272.0–296.5) among females. Respective estimates for the period 2004–2008 were 351.9 (337.5–366.8) and 335.3 (321.3–349.9).

#### Prevalence

The Poisson model with the best fit for the prevalent cases stratified by region and age (Poisson model M3) included region and age as independent predictors (data on model selection not shown). Prevalence estimates by region and age predicted from the Poisson model are given in Table 4.

**Table 4:** Prevalence of T1DM in 2008 by region and age estimated from Poisson model M3.

				Prevalence (95% CI) per 100,000 persons			
Region	Period	N	Person-years	0–4 years	5–9 years	10–14 years	0–14 years*
**North Rhine-Westphalia**	2008	4055	2,553,165	35.8 (32.9–39.0)	154.9 (148.5–161.6)	261.7 (253.1–270.5)	150.8 (147.1–154.6)
**Baden-Wuerttemberg**	2008	2186	1,571,620	31.4 (28.7–34.3)	136.0 (129.4–142.9)	229.7 (220.2–239.6)	132.4 (128.4–136.5)
**Saxony**	2008	550	454,198	30.5 (27.4–34.1)	132.1 (121.7–143.4)	223.2 (206.1–241.7)	128.6 (121.8–135.8)
**Germany**	2008	17088	11,138,806	35.2 (33.4–37.0)	152.1 (137.0–169.0)	257.0 (232.1–284.5)	148.1 (138.2–158.7)

*age-standardized, Poisson model M3 included region and age on 31.12.2008 (0–4, 5–9, 10–14 years) as independent variables, pseudo-R^2^ = 0.999

The predicted estimates are in excellent agreement with the observed prevalence (Table 4, pseudo-R^2^ = 0.999).

Predicted age-specific national prevalence estimates are also given in Table 4. The model-based national prevalence in the age group of 0–14 years was 148.1. The prevalence showed significant variation between regions (p<0.001) (Table 4). The predicted age-standardized prevalence in Saxony and Baden-Wuerttemberg was significantly lower than the national prevalence and the prevalence in North Rhine-Westphalia (p<0.001).

The best fitting model for prevalence data stratified by region, sex and age group (Poisson model M4) included region, sex, age group, and a term for sex by age group interaction as independent predictors. Sex-specific, age-standardized national and regional prevalence estimates derived from this Poisson model are given in Table 5. For all regions, the model-based prevalence was comparable between males and females.

**Table 5:** Age-standardized prevalence in 2008 by region and sex predicted from Poisson model M4.

		Prevalence* (95% CI) per 100,000 person-years	
Region	Period	Male	Female
**North Rhine-Westphalia**	2004–2008	151.2 (146.9–155.7)	151.2 (146.8–155.8)
**Baden-Wuerttemberg**	2004–2008	132.4 (128.0–136.9)	132.4 (127.9–137.0)
**Saxony**	2004–2008	126.0 (120.1–132.1)	126.0 (120.0–132.2)
**Germany**	2004–2008	148.0 (138.7–157.9)	148.0 (138.6–158.0)

* age-standardized; population 0–14 years, Poisson model M4 included region, sex, age on 31.12.2008 (0–4, 5–9, 10–14 years) and a term for age by sex interaction as independent variables, pseudo-R^2^ = 0.998

## Discussion

This is the first study to pool prospectively national incidence and prevalence data for the ages 0–4 years and regional data for the age 0–14 years from independent registries from 3 federal states with a high coverage (41%) of the child population in Germany in order to estimate the national incidence rate and prevalence.

The different regional incidence rates in Germany fit into/are in accordance with the observed West-East gradient in Europe [6]. Moreover, prevalence estimates for overweight in children [24, 25] support overweight–in accordance with the overload hypothesis [26]–as a potential factor for higher incidence rates in NW than in Saxony. The EURODIAB project reported also a West-East gradient with very low incidence rates in the eastern European countries, but a relatively high annual increase since 1989 in these regions (Lithuania: 10 per 100,000 py, Hungary, Rumania and Slovenia: 11 per 100,000 py, Poland: 13 per 100,000 py [6]). The causes underlying these patterns still remain to be elucidated. Most recent data from Sweden, Finland and Norway suggest that there may be a levelling-off or even a reversal of the increasing trend in most recent years [5, 6].

As might have been expected, the estimated incidence rates for Germany compared to the rest of Europe range between the rate in Scandinavian countries and Southern Europe [5]. Within Europe, the highest incidence rates in the ages 0–14 years were found in northern regions with a current incidence rate from 32 to 58 per 100,000 per year in Sweden, Norway and Finland [3–5]. Lower incidence rates are reported for Southern Europe (i. e. Montenegro: 17.5 per 100,000 py and Spain: 12 per 100,000 py [5]), suggesting a potential north-south gradient. An expected moderate incidence rate was also found in continental Italy (15 per 100,000 py), whereas an unexplained high rate was observed on the island of Sardinia (41 per 100,000 py [7]).

With an estimated national incidence rate of 22.9/100,000 py (2004–2008), Germany is a country with a higher risk for childhood T1DM compared to many other countries around the world [27]. The average annual increase in T1DM in Germany seems to affect all age groups equally. According to international data, there is no conclusive relation between sex and incidence rate. Higher incidence rates among the male population were found in countries with high T1DM incidence rates, higher incidence rates among the female population in countries with relatively low incidence rates [28].

Our model-based estimate confirms the international trend of an increasing incidence rate in T1DM. The national estimates for Germany suggest an annual increase in T1DM incidence of 3.4%. A worldwide average annual increase in T1DM incidence rate of 3–4% during past decades has been reported [2, 5]. But rates of increase vary between regions and time-periods [5]. Regarding a projection of the incidence rate in Germany for the next decades, we refer to a recent paper by Ehehalt et al. 2010 [12].

There are many theories dealing with the global increase of T1DM incidence rates. In general, a multifactorial process with various environmental triggers is assumed for the aetiology of T1DM [29]. As mentioned above, the overload hypothesis claims a higher risk for T1DM due to overweight, physical inactivity, and stress [26].

### Strengths and Limitations of the Study

There is no nationwide registration of childhood T1DM (0–14 years) in Germany, as yet. Consequently, only a model-based approach can provide reasonable national estimates. The strengths of our study are that estimates are based on a large proportion of the childhood population of Germany (0–4 years whole population 5–14 years 41% of German population), that registries used had high ascertainment, and we applied standard statistical procedures as well as detailed statistical modelling, using widely recognized criteria for model selection.

One shortcoming of the study is that we could not use absolutely up-to date data for estimation due to restrictions in use of the registered data. Nevertheless, the presented national estimates are interesting novel information for Germany, because these are the first estimates based on pooling of data from 3 regional registers in the age group 0 to 14 years and a national register for the age group 0 to 4 years. A further limitation is that national estimates based on only 3 federal states may be biased due to regional differences in incidence rates. However, since our model-based approach used nationwide data for 0–4 and regional data for 0–14 years, the estimation approach is in fact based on the assumption that incidence rate ratios or prevalence ratios between age groups do not seriously depend upon the region, as suggested by the fact that the age by region interaction was not significant in any of the models (data not shown). Therefore, our national estimates can be assumed to approximate the actual national data quite well.

Prevalent cases were taken from incidence registries, thus mortality was not taken into account. However, since mortality from childhood T1DM is very low in Germany, there is only a small upward-bias in our estimates and results should not be seriously affected by mortality [30].

## Conclusions

With an estimated national incidence rate of 22.9/100,000 py (0–14 years, 2004–2008) and an estimated national prevalence of 148.1/100,000 (0–14 years, on 31/12/2008), the risk for childhood T1DM in Germany is higher than in many other countries around the world. The annual increase of 3.4% affects all age groups and regions, but reasons for regional differences in incidence rates and its trends still have to be elucidated. Due to the high coverage of the German childhood population in our study, our estimates are expected to be quite reliable. Although this work–using for the first time national data in the age group 0–4 years and regional data in the age group 0–14 years from 3 federal states (North Rhine-Westphalia, Baden-Wuerttemberg and Saxony)–provides national estimates, a nationwide registration of T1DM cases in children under 15 years should still be aimed for in the longer term.
